# Identification of Borderline Personality Disorder in Adolescents: Psychometric Properties and Diagnostic Efficiency of a Juvenile Version of the Impulsivity and Emotion Dysregulation Scale (IES‐27‐J)

**DOI:** 10.1002/jclp.23792

**Published:** 2025-03-25

**Authors:** Maria Brede, Brigitte Dippold, Stephan Bender, Christoph Kröger, Maya Krischer

**Affiliations:** ^1^ Department of Clinical Psychology and Psychotherapy University of Hildesheim Hildesheim Germany; ^2^ Department of Child and Adolescent Psychiatry University Hospital of Cologne Cologne Germany

**Keywords:** Borderline Personality Disorder, diagnostic efficiency, emotional dysregulation, impulsivity, psychometric properties, reliability

## Abstract

**Objective:**

The diagnostic efficiency of screening instruments for adolescents with Borderline Personality Disorder (BPD)—that are applicable to its new classification in DSM‐5 and ICD‐11—has not yet been sufficiently studied.

**Methods:**

We examined the reliability and diagnostic efficiency of the juvenile version of the *Impulsivity and Emotion Dysregulation Scale* (IES‐27‐J) in a German‐speaking sample of inpatient 12–19‐year‐old adolescents (*N* = 220, including *n* = 88 with BPD and *n* = 132 with other mental disorders). Using a receiver operating characteristic (ROC) analysis, optimal cutoff values were determined for this self‐report instrument. Analyses were conducted for two different diagnostic thresholds with at least four and five BPD diagnostic criteria, respectively, in accordance with the semi‐structured clinical interview *International Personality Disorder Examination* (IPDE).

**Results:**

Results indicate that the IES‐27‐J is a reliable and valid instrument with moderate to high discriminative ability (areas under the curve [AUC] = 0.77 and 0.80, respectively). Using the preferred cutoff values, sensitivity (71% and 83%) and specificity (76% and 67%) turned out to be moderate.

**Conclusion:**

The application of the IES‐27‐J can be considered favorable in a two‐stage approach, using a lower cutoff value in a first step to miss fewer patients with BPD, and conducting a clinical interview in a second step to confirm the diagnosis. More studies in different settings, including direct comparisons with other screening instruments, are necessary to further assess the clinical utility of the IES‐27‐J.

## 1 Introduction

Borderline personality disorder (BPD) is a severe mental disorder characterized by interpersonal difficulties, identity instability, as well as emotional dysregulation and high impulsivity. The high prevalence rate of suicidality and self‐harm, particularly among juveniles with BPD, poses significant challenges for the healthcare system (Gunderson et al. 2018). To prevent a chronic loss of psychosocial functioning, as well as the maintenance and exacerbation of BPD symptoms, experts affirm that early identification and subsequent disorder‐specific therapy are increasingly crucial (Birkhölzer et al. 2020; Chanen et al. 2017). Numerous reviews and meta‐analyses in recent years suggested that BPD is as valid and reliable a diagnosis in adolescence as it is in adulthood, based on similarity in prevalence (Ellison et al. 2018), in phenomenology (Winsper et al. 2016), in functional impairment and stability (Alvarez‐Tomás et al. 2017; Chanen et al. 2004; Videler et al. 2019). This has led to the new classification of personality disorders and BPD in ICD‐11 (World Health Organization 2019), which recommends a dimensional diagnostic approach, removes previous age limits, and suggests diagnosing personality disorders from the age of 12 (Schmeck and Birkhölzer 2021).

Despite these advances, the psychometric properties and particularly the diagnostic efficiency of BPD‐specific screening instruments have not been sufficiently investigated for adolescents. Although there are now several studies examining the diagnostic efficiency of BPD‐specific screening instruments for adolescents, most of these studies (Chanen et al. 2008; Chang et al. 2011; Noblin et al. 2014; Sharp et al. 2014) have methodological limitations (see Table S1 for an overview). First of all, most studies do not report whether the required sample size was calculated a priori. Considering the base rate of BPD in the sample as well as the (estimated) sensitivity and specificity of the instrument, calculations should be obtained individually for each study (Akoglu 2022). An examination of the recommended sample sizes according to Hajian‐Tilaki (2014) showed that the sample sizes of four studies were smaller than recommended (*N* = 51–118 < 204 at least). Therefore, an increased marginal error may have affected the estimation of the diagnostic efficiency indices (Hajian‐Tilaki 2014). However, there is one exception that might be a benchmark for the present study. Based on a sample size of *N* = 371 inpatients and a BPD rate of 33%, Sharp et al. (2014) calculated the following for the 11‐item version of the Borderline Personality Disorder Features Scale for Children (BPFS‐C): an area under curve of AUC = 0.80, a sensitivity of 74%, and a specificity of 71%. Secondly, three of four studies (Chang et al. 2011; Noblin et al. 2014; Sharp et al. 2014; see Table S1) do not report Cohen's κ as a measure of agreement between the screening instrument and the reference instrument, nor do they report cross‐tables that would allow this value to be determined. Compared to many other indices, this Cohen's κ corrects for the random factor and allows for a stronger weighting of sensitivity and specificity (Dhamnetiya et al. 2021; Streiner 2003). Thus, the weighted Cohen's κ should be considered when selecting the optimal cutoff value. Third, in most studies, the choice of the optimal cutoff value is based on a balance between sensitivity and specificity (e.g., the intersection of the two). One of the studies (Chanen et al. 2008) even makes a purely graphical (i.e., AUC‐curve‐based) decision for a cutoff only. Many studies do not report indices of diagnostic efficiency for different possible cutoffs, as suggested by Zimmerman and Balling (2021).

The juvenile version (*IES‐27‐J*; Kröger et al. 2017) of the Impulsivity and Emotion Dysregulation Scale (*IES‐27*; Kröger and Kosfelder 2011) with 27 items has proven to be a reliable and valid instrument in two initial studies (Kröger et al. 2017). For example, the internal consistency value was α = 0.95 for the IES‐27‐J in two samples. The test‐retest reliability was excellent (ρ = 0.97). Juveniles with BPD achieved significantly higher sum scores than patients with a depressive or conduct disorder (Kröger et al. 2017) and patients with other mental disorders characterized by impulsivity and emotion dysregulation (e.g., attention deficit hyperactivity disorder and eating disorders; Dreyße et al. 2021), which can be an indication of the discriminative validity of the IES‐27‐J.

However, results on the diagnostic efficiency of the IES‐27‐J are still to be determined. Therefore, this study aims at a further evaluation based on various psychometric parameters. First, item characteristics and reliability indices were calculated to test the replicability of the results in a different sample. Second, the diagnostic efficiency of the IES‐27‐J was determined using a ROC analysis. Following the recommendation of Zimmerman and Balling (2021), we provided various indices (i.e., sensitivity, specificity, [weighted] positive and negative predictive values, Youden's index J, overall correct classification rate and [weighted] Cohen's κ) for several cutoff points and preferred to give sensitivity more consideration when choosing the optimal cutoff for the IES‐27‐J. Analyses were performed based on two different diagnostic thresholds. Those adolescents who met at least four diagnostic criteria according to the IPDE interview were included in the BPD group; then, in a second run, adolescents who met at least five criteria were included in the BPD group. The decision to perform analyses based on full diagnostic criteria versus at least four criteria (“subthreshold BPD”) is based on the results of Kaess et al. (2017) and Thompson et al. (2019), who showed high impairment in adolescents even when fewer BPD criteria were met. The authors concluded that lowering the cutoff may also be useful in child and adolescent psychiatric care to identify at‐risk adolescents early and provide timely interventions (Chanen et al. 2017; Kaess et al. 2017).

## 2 Materials and Methods

### 2.1 Procedure

The present study is based on a subset of data from a study conducted by the University Hospital of Cologne on the efficacy of specialized treatment approaches for adolescents with personality disorders. The study has been approved by the Ethics Committee of the University Hospital of Cologne and has been registered in the German Register of Clinical Trials (DRKS00010557; Registration date: August 27, 2020). Data were collected consecutively from September 2017 to November 2022 in the Department of Child and Adolescent Psychiatry at the University Hospital of Cologne. After receiving verbal and written information about the study, all patients registered for treatment in any of the wards were invited to participate. All admitted patients had to agree to and to sign a treatment contract before entering the program. Inclusion criterion for the efficacy study was consent for voluntary participation by the adolescent and at least one legal guardian (if the adolescent was younger 16 years of age). There were no further exclusion criteria.

After the sociodemographic data was collected in a clinical anamnestic interview, participants were asked to complete the IES‐27‐J. To identify personality disorders, the International Personality Disorder Examination (IPDE) was conducted with all adolescents. Each interviewer had a university degree in psychology or education and was in training to become a child and adolescent psychotherapists. All of them underwent a 10‐h training in administration and scoring of the IPDE, conducted M. Krischer, the principal investigator, who was proven to be a reliable interviewer by A. Loranger in 2001. M. Krischer developed a demonstration IPDE tape featuring a juvenile patient, to be used as a rating basis. All interviewers had to achieve 80% reliability with the professional rating of the demo tape to conduct interviews in the study. Both the IPDE and the questionnaire were used in the German language versions. The presence of other mental disorders, except personality disorders, was assessed via clinical interviews carried out by licensed psychologists and psychiatrists in accordance with ICD‐10.

### 2.2 Sample Size Calculation

The required sample size was determined using the formulas of Negida et al. (2019) and Hajian‐Tilaki (2014; see Calculation S2). As no sensitivity and specificity values were previously available for the IES‐27‐J, we used the values from the study by Chang et al. (2011) on the Borderline Personality Disorder Features Scale for Children (BPFS‐C; see Table S1). This study was suitable for an estimate of the required sample size because the instrument has a similar number of items, and the characteristics of the sample (i.e., psychiatric inpatients, high prevalence of BPD, high proportion of co‐morbidity) were similar to those of our target sample. The calculation resulted in a required sample size of at least *N* = 204, which we used as an approximate guideline. Considering further co‐morbid mental disorders as distractors, the sample size might be larger for ROC analyses.

### 2.3 Participants

The sample originally consisted of *N* = 244 children and adolescents who opted for treatment in one of the inpatient or day hospital units of the Department of Child and Adolescent Psychiatry at the University Hospital of Cologne. All participants signed a treatment contract and received psychiatric outpatient care before seeking inpatient specialized treatment. There were no differences between day‐clinic and inpatients in terms of severity of illness, self‐harm and suicidal tendencies. Admission to the wards required a willingness to stay overnight. All participants and at least one legal guardian gave written consent for voluntary participation after being personally informed about the study. None of them received financial compensation. Twenty‐two subjects were excluded due to missing IPDE interviews, and two additional subjects were excluded because they were missing more than 33% of the item scores on the IES‐27‐J. The excluded subjects did not differ from the remaining sample in terms of IES‐27‐J scores (*t*(234) = −0.50, *p* = 0.62), age (*t*(242) = −0.11, *p* = 0.91), gender (χ²(3) = 0.54, *p* = 0.91) or other socio‐demographic variables (living situation: χ²(12) = 9.32, *p* = 0.78; marital status of parents: χ²(4) = 0.89, *p* = 0.93; current school: χ²(6) = 13.45, *p* = 0.04, α_adj_ = 0.008).

The final sample consisted of *N* = 220 children and adolescents, of whom 82% (*n* = 180) were female, 17% (*n* = 38) were male, and 1% (*n* = 2) were gender diverse. The mean age was 15.8 years (SD = 1.4, range: 12–19 years). Table 1 provides an overview of the sociodemographic characteristics of the total sample and the different disorder groups.

**Table 1:** Sociodemographic characteristics.

	Total (*N* = 220)	BPD (*n* = 88)	Other PDs and MDs (*n* = 132)
Age in years: *M (SD)*	15.8 (1.4)	15.7 (1.4)	15.9 (1.3)
Gender (m/f/d): %	82/17/1	91/8/1	76/23/1
Living situation: *n* (%)			
With both parents	85 (39%)	36 (41%)	49 (37%)
With one parent	81 (37%)	27 (31%)	54 (41%)
With foster, surrogate or Adoptive parents	11 (5%)	6 (7%)	5 (4%)
In inpatient youth care	26 (12%)	12 (14%)	14 (11%)
Missing data	17 (8%)	7 (8%)	10 (8%)
Marital status of parents: *n* (%)			
Married	102 (46%)	41 (47%)	61 (46%)
Divorced	59 (27%)	27 (31%)	32 (24%)
Living apart	41 (19%)	11 (12%)	30 (23%)
Other relationship status	15 (7%)	6 (7%)	8 (6%)
Missing data	3 (1%)	3 (3%)	1 (1%)
Current school: *n* (%)			
Secondary general school^a^	21 (10%)	12 (14%)	9 (7%)
Secondary school^b^	44 (20%)	16 (18%)	28 (21%)
Comprehensive school^c^	54 (24%)	24 (27%)	30 (23%)
Academic secondary school^d^	86 (39%)	30 (34%)	56 (42%)
Special needs education school^e^	6 (3%)	3 (3%)	3 (2%)
Other type of school	6 (3%)	1 (1%)	5 (4%)
Missing data	3 (1%)	2 (2%)	1 (1%)

Abbreviations: d = gender divers, f = female, m = male.

^a^ Hauptschule.

^b^ Realschule.

^c^ Gesamtschule.

^d^ Gymnasium.

^e^ Förderschule.

Among the 220 patients, 40% (*n* = 88) met at least five criteria of BPD and 36% (*n* = 79) reported criteria for at least one other personality disorder (other PDs) as assessed by the IPDE. Table S3 shows the frequency of the different PDs according to DSM‐IV in the total sample and for each group of adolescents with and without BPD. The remaining 24% (*n* = 53) of adolescents had at least one mental disorder other than PD (other MDs), most prevalently depressive disorders. No age difference between the disorder groups were found (*t*(218) = 1.21, *p* = 0.23); however, there were more females with BPD (91%) compared to the groups of adolescents with other disorders (76%) (χ²(3) = 11.02, *p* = 0.01, Cramer's *V* = 0.22).

If the IPDE threshold was lowered so that adolescents meeting only at least four criteria are also included in the BPD group, the size of the disorder groups changes as follows: 62% (*n* = 136) of adolescents are in the BPD group, 24% (*n* = 53) are in the other PD group, and 14% (*n* = 31) are in the other MD group.

### 2.4 Measures

Juvenile version of the *Impulsivity and Emotion Dysregulation Scale* (IES‐27‐J; Kröger et al. 2017). The IES‐27‐J is a self‐report questionnaire for adolescents aged 13 to 18 years that asks about the frequency of various BPD‐specific experiences and behaviors related to impulsivity and emotional instability over the past month. A total of 27 items are scored on a five‐point Likert scale (0 = *not at all*, 1 = *1–2 times*, 2 = *3–10 times*, 3 = *daily* and 4 = *several times daily*). The total score is calculated by adding the individual item scores, with a maximum of 108. The instrument is based on seven of the nine BPD criteria, with the exception of criteria 7 (emptiness) and 9 (paranoid ideation and dissociation). The items of these criteria were eliminated based on factor‐analyses of data in previous versions. A confirmatory factor analysis of the IES‐27‐J revealed a general factor and three specific factors (Emotional Dysregulation, Self‐injurious and Suicidal Behavior, and Interpersonal Difficulties) (Dreyße et al. 2021). The structure of the scale thus reflects the three central BPD‐dimensions (e.g., Giesen‐Bloo et al. 2010). Initial studies evaluating the juvenile version IES‐27‐J revealed very high reliability values (Cronbach's α = 0.95, Guttman's split‐half coefficients λ = 0.97, test‐retest reliability ρ = 0.93). Furthermore, mid‐range difficulty indices and predominantly good‐to‐excellent part‐whole corrected item‐scale correlation coefficients (0.39 ≤ *r*_*itc*_ ≤ 0.81) indicated the strong discriminative ability of the items (Kröger et al. 2017).

*International Personality Disorder Examination* (IPDE; Loranger 1997; German version: Mombour et al. 1996). The IPDE is a semi‐structured clinical interview designed to assess personality disorders according to DSM‐IV and ICD‐10. Each diagnostic criterion is scored as either 0 = *not met*, 1 = *markedly present*, or 2 = *present*. For the categorical diagnosis of BPD, interrater reliability value was rated as good, with *κ* = 0.80 and test‐retest reliability value was rated as moderate with *κ* = 0.70. The internal consistency of the BPD items was found to be Cronbach's α = 0.64 (Carcone et al. 2015).

### 2.5 Data Analyses

Data analyses were performed using SPSS 28.0 statistic software for Windows 11. An analysis of the missing values of the IES‐27‐J items was conducted. Little's MCAR test was not significant (χ²(25926) = 12415.744, *p* = 1.00), indicating that the missing values were missing completely at random (MCAR). Multiple imputation was then used to estimate the missing data, as this procedure leads to approximately unbiased estimates of standard errors and test statistics (Rubin 1987). For further calculations, missing values were replaced by the point estimate, which is the arithmetic mean of the multiple imputed values, according to Rubin (1987).

#### 2.5.1 Item Characteristics and Reliability Indices

The item analysis included the determination of the difficulty index *p*_*m*_ and the part‐whole corrected item‐scale correlation coefficients *r*_*itc*_ of the items. Item difficulty indices should be 0.20 ≤ *p*_*m*_ ≤ 0.80 to assume a high ability of the items to discriminate between subjects with high or low trait expression. Part‐whole corrected item‐scale correlation coefficients should be *r*_*itc*_ ≥ 0.30 to assume a good discriminative ability of the items (Moosbrugger and Kelava 2020). The internal consistency was determined using Cronbach's α and McDonald's ω coefficients. According to Waller (2008), the presence of commingled samples can lead to an overestimation or underestimation of Cronbach's α. For this reason, we corrected values using Formula 16 in Waller (2008). For both coefficients, values < 0.80 were considered small, values between 0.80 and 0.90 were considered moderate to good, and values > 0.90 were considered excellent (Schmidt‐Atzert and Amelang 2012).

#### 2.5.2 Diagnostic Efficiency

The ROC analysis is a well‐established method for evaluating the ability of biomarkers or measures to discriminate individuals who have a disease or disorder from those who do not (Dhamnetiya et al. 2021). In this analysis, the classifications of the IES‐27‐J (BPD present vs. BPD not present) were compared with the classifications or diagnoses according to the IPDE as a reliable and sufficiently validated reference instrument. ROC curves were plotted to visualize the trade‐off between sensitivity or true positive rate and (1‐specificity) or false positive rate of the IES‐27‐J across a series of cutoff values. In addition to AUC, sensitivity (SEN), specificity (SPE), positive predictive values (PPV), and negative predictive values (NPV) were determined for various cutoff values. Because the predictive values are highly dependent on the prevalence rate, the weighted PPV_w_ and NPV_w_ were additionally calculated using Formulas 15 and 17 in Streiner (2003). Specifically, the PPV_w_ and NPV_w_ were determined for a prevalence rate of 25% to assess the performance of the measure in samples with a lower prevalence. As possible criteria for the selection of the optimal cutoff value, four additional indices were calculated. In general, the cutoff value that maximizes the respective criterion is preferred (Dhamnetiya et al. 2021). First, the Youden's index (*J* = SEN + SPE – 1; Youden 1950) was calculated. For a screening instrument, *J* should be > 0.50 (Youden 1950). As another possible criterion, the overall correct classification rate (OCC), i.e., the proportion of correct decisions (Streiner 2003), was determined. Since the index *J* and the OCC do not take into account a randomly occurring correct classification such that the classification accuracy of the IES‐27‐J may be overestimated, Cohen's κ was additionally calculated with Formula 9 in Streiner (2003). To account for the greater importance of SEN in a screening instrument, this coefficient was additionally weighted (75% SEN, 25% SPE) (κ_w_; Streiner 2003). The choice of the preferred cutoff value was made on the basis of a joint consideration of the four criteria. Since the health and economic costs of late or missed detection of BPD are very high (Chanen et al. 2017), the sensitivity of the instrument was given a higher weight in this evaluation.

## 3 Results

### 3.1 Item Characteristics and Reliability Indices

The items and their characteristics are displayed in Table 2. The item difficulty indices were mostly in the mid‐range (0.20 ≤ *p*_*m*_ ≤ 0.80), which indicates a high discriminative ability of the items. As expected, lower index values were found for items that ask about the frequency of suicidal and severe self‐injurious behavior (items 7, 18, and 25). Overall, there was a wide distribution of low and high difficulty indices (0.05 ≤ *p*_*m*_ ≤ 0.62), which suggests that the items are able to discriminate across a wide range of symptom severity. The part‐whole corrected item‐scale correlation coefficients were predominantly in the good to excellent range (0.28 ≤ *r*_*itc*_ ≤ 0.74), which indicates a good discriminative ability of the items.

**Table 2:** IES‐27‐J items and item characteristics.

No.	Items: In the last month …	*M*	*SD*	*p*_*m*_	*r*_*itc*_
1.	… I was afraid of losing control over my feelings	1.97	1.28	0.49	0.67
2.	… I hurt myself by consciously knocking my head, my arm or other parts of my body against something	1.02	1.07	0.26	0.54
3.	… I hurt myself by superficially cutting or scratching myself	1.08	1.01	0.27	0.46
4.	… I was afraid of losing control of my actions	1.67	1.20	0.42	0.63
5.	… I was so angry that I could hardly control myself	1.10	1.07	0.28	0.61
6.	… I had eating binges	1.09	1.12	0.27	0.43
7.	… I prepared to attempt suicide	0.31	0.55	0.08	0.42
8.	… my feelings went up and down like a roller coaster	2.40	1.27	0.60	0.67
9.	… I acted very erratic	1.50	1.17	0.38	0.62
10.	… I had an argument with someone	1.61	1.03	0.40	0.52
11.	… I had fantasies of revenge	0.70	1.04	0.18	0.40
12.	… I was thinking of means and ways to kill myself	0.95	1.05	0.24	0.45
13.	… my feelings changed rapidly	2.40	1.26	0.60	0.68
14.	… my relationships were constantly up and down	1.37	1.22	0.34	0.65
15.	… I did something without considering the consequences for me or for someone else	1.02	1.03	0.25	0.60
16.	… I thought about killing myself	1.62	1.35	0.40	0.47
17.	… I experienced intense hatred	1.54	1.18	0.38	0.55
18.	… I hurt myself by burning myself	0.20	0.50	0.05	0.35
19.	… I was overwhelmed by my feelings	2.05	0.50	0.51	0.74
20.	… I was very tense and under high pressure	2.48	1.04	0.62	0.62
21.	… I hurt another person's feelings, which I regretted afterwards	0.46	0.79	0.12	0.46
22.	… I had trouble with other people	0.75	0.85	0.19	0.41
23.	… I consoled myself with fantasies of suicide	0.94	1.07	0.23	0.56
24.	… my emotions were muddled	2.07	1.26	0.52	0.67
25.	… I hurt myself by cutting myself deeply	0.41	0.65	0.10	0.36
26.	… I was angry	2.00	1.04	0.50	0.58
27.	… I vomited	0.57	0.91	0.14	0.28

*Note: p*_*m*_ = difficulty index. *r*_*itc*_ = part‐whole corrected item‐scale correlation coefficient.

For the total score of the IES‐27‐J, the internal consistency appeared to be excellent, with a corrected Cronbach's α = 0.93 and McDonald's ω = 0.93 for both IPDE‐thresholds. Eliminating items with low item–scale correlation coefficients (*r*_*itc*_ < 0.30) or low difficulty indices (*p*_*m*_ < 0.20) did not improve internal consistency.

### 3.2 Diagnostic Efficiency

**Figure 1:**
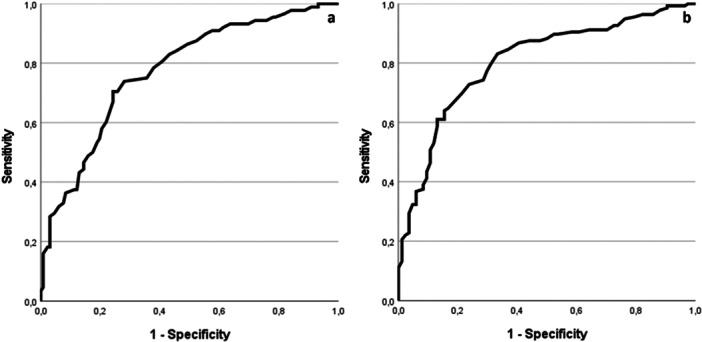
ROC curves: Five‐criteria threshold (a) and four‐criteria threshold (b).

Based on the two IPDE‐thresholds, (i.e., at least five or at least four BPD criteria), areas under the curves were AUC = 0.77 (95% CI [0.71, 0.84]) and AUC = 0.80 (95% CI [0.75, 0.86]), respectively. According to Dhamnetiya et al. (2021), these values indicate a moderate to high discriminative ability of the IES‐27‐J. Figure 1 shows the ROC curves for both IPDE‐thresholds. Table 3 presents an excerpt of possible cutoff values and associated diagnostic efficiency characteristics.

**Table 3:** Possible cutoff points and related diagnostic efficiency characteristics.

	Four‐criteria IPDE threshold										Five‐criteria IPDE threshold									
Cut‐off	SEN	SPE	PPV	PPV_w_	NPV	NPV_w_	*J*	OCC	κ	κ_w_	SEN	SPE	PPV	PPV_w_	NPV	NPV_w_	*J*	OCC	κ	κ_w_
≥ 28	0.85	0.63	0.79	0.43	0.72	0.92	0.48	0.76	0.49	0.46	0.88	0.48	0.53	0.36	0.85	0.92	0.35	0.64	0.32	0.25
**≥ 29**	**0.83**	**0.67**	**0.80**	**0.45**	**0.71**	**0.92**	***0.50***	***0.77***	***0.50***	**0.49**	0.86	0.51	0.54	0.37	0.85	0.92	0.37	0.65	0.34	0.27
≥ 30	0.79	0.69	0.81	0.46	0.67	0.91	0.48	0.75	0.48	0.49	0.84	0.54	0.55	0.38	0.84	0.91	0.39	0.66	0.36	0.30
≥ 31	0.77	0.70	0.81	0.46	0.66	0.90	0.47	0.75	0.47	0.48	0.83	0.57	0.56	0.39	0.83	0.91	0.40	0.67	0.37	0.31
≥ 32	0.74	0.71	0.81	0.46	0.63	0.89	0.46	0.73	0.45	0.47	0.81	0.59	0.57	0.40	0.82	0.90	0.40	0.68	0.37	0.32
≥ 33	0.73	0.76	0.83	0.50	0.63	0.89	0.49	0.74	0.47	0.51	0.78	0.62	0.58	0.41	0.81	0.90	0.41	0.69	0.38	0.34
≥ 34	0.70	0.79	0.84	0.52	0.62	0.89	0.48	0.73	0.46	0.51	0.75	0.64	0.58	0.41	0.79	0.89	0.39	0.69	0.38	0.34
≥ 35	0.65	0.83	0.86	0.56	0.59	0.88	0.48	0.72	0.45	0.53	0.74	0.72	0.64	0.47	0.81	0.89	0.46	0.73	0.45	0.42
≥ 36	0.64	0.85	0.87	0.58	0.59	0.88	0.48	0.72	0.45	0.53	0.73	0.73	0.64	0.47	0.80	0.89	0.45	0.73	0.44	0.42
≥ 37	0.61	0.85	0.86	0.57	0.57	0.87	0.46	0.70	0.42	0.51	0.71	0.74	0.65	0.48	0.79	0.88	0.45	0.73	0.44	0.42
**≥ 38**	0.61	0.87	0.88	0.61	0.58	0.87	0.48	0.71	0.44	*0.54*	**0.71**	**0.76**	**0.66**	**0.49**	**0.79**	**0.89**	***0.46***	***0.74***	***0.46***	***0.44***
≥ 39	0.59	0.87	0.88	0.60	0.57	0.86	0.46	0.70	0.41	0.52	0.67	0.76	0.65	0.48	0.78	0.87	0.43	0.72	0.43	0.42

*Note:* Bold marked values = preferred cutoff points and related indices. Values in italics = maxima of the indices used as criteria for the selection of the optimal cutoff. SEN = sensitivity. SPE = specificity. PPV = positive predictive value. PPV_w_ = positive predictive value which was weighted with a prevalence rate of 25%. NPV = negative predictive value. NPV_w_ = negative predictive value which was weighted with a prevalence rate of 25%. *J* = Youden's index. OCC = overall correct classification rate. κ = Cohen's Kappa. κ_w_ = Cohen's Kappa which was weighted for sensitivity (75%).

#### 3.2.1 Cutoff Value Using Four BPD Criteria

Both the maximum *J* and the maximum κ suggested an optimal cutoff value of ≥ 29. For this cutoff value, the OCC was also the highest at 77%. 83% of patients with BPD (SEN) and 67% of individuals without BPD (SPE) were correctly identified using this cutoff value. The probability that individuals classified as BPD patients in fact fulfilled the criteria for this disorder was 80% (PPV), whereas the likelihood that the disorder was not actually present when no BPD was classified was 71% (NPV). Taking into account a lower prevalence rate of 25%, the PPV_w_ and NPV_w_ were 45% and 92%, respectively. The index *J* of 0.50 calculated for this cutoff value corresponded closely to the recommended amount for a screening measure (*J* > 0.50; Youden 1950). The κ of 0.50 and κ_w_ of 0.54 indicate a moderate agreement between the IES‐27‐J classification and the IPDE classification. The application of the preferred cutoff value resulted in 10% (*n* = 23) false‐negative cases and 13% (*n* = 28) false‐positive cases (see Table S4).

#### 3.2.2 Cutoff Value Using Five BPD Criteria

Using the five‐criteria IPDE‐threshold, the four indices consistently suggested an optimal cutoff value of ≥ 38 for the IES‐27‐J., resulting in OCC = 74%. As displayed in Table 3, the ROC characteristics were in a similar amount as reported for the cutoff value using four BPD criteria. The index *J* of 0.46 narrowly missed the recommended level. The agreement between the classifications of both instruments was moderate, as indicated by κ = 0.46 and κ_w_ = 0.44. Using the cutoff value of ≥ 38, 12% (*n* = 26) were classified as false‐negative and 14% (*n* = 32) as false‐positive cases (see Table S5).

## 4 Discussion

The purpose of the study was the evaluation of the IES‐27‐J screening instrument in terms of item characteristics, reliability and diagnostic efficiency.

The item difficulty indices, most of it fell in the mid‐range (0.12 ≤ *p*_*m*_ ≤ 0.62), along with the predominantly good to excellent part‐whole corrected item‐scale correlation coefficients (0.28 ≤ *r*_*itc*_ ≤ 0.74), demonstrate that the items reliably discriminate between patients with high and low symptom severity. As expected, items assessing the frequency of suicidal and severe self‐injurious behaviors yielded lower difficulty scores and indicate limited discriminative ability. However, eliminating these items did not improve internal consistency, so they may still provide valuable diagnostic cues and should be retained. The present study also showed excellent internal consistency values (corrected Cronbach's α = 0.93; McDonald's ω = 0.93), indicating that the items are highly related to each other and reliably measure the same underlying homogenous construct. This finding is consistent with a previous study (Kröger et al. 2017), which reported a high internal consistency value (α = 0.95) and a single‐factor structure. Discrepancies with the results of this study that appeared in the difficulty indices of some items could be attributed to differences in sample characteristics. Thus, the higher difficulty of items measuring emotional dysregulation in the current sample may be explained by the higher proportion of adolescents with BPD. The corrected value for the internal consistency of the IES‐27‐J can be rated as similar to or above values in comparison to other screening instruments (see Table S1 for indices of other instruments).

ROC analysis showed a moderate to high discriminative ability for the IES‐27‐J. Based on the two IPDE‐threshold, the diagnostic efficiency indices suggest optimal cutoff values of ≥ 29 and ≥ 38 for the IES‐27‐J, respectively. Furthermore, ROC characteristics were appropriate to good for a self‐rated screening measure. However, the values for κ and κ_w_ correspond to a moderate agreement between the IES‐27‐J classification and the actual diagnosis according to the IPDE for both thresholds. The recommended Youden's index for a screening instrument of *J* > 0.50 was narrowly missed, with *J* = 0.50 and 0.46, respectively. Thus, the IES‐27‐J failed to identify 17% and 28% of adolescents with BPD for these respective thresholds. Compared to the diagnostic efficiency indices obtained for other BPD‐specific screening instruments in samples of adolescents (see Table S1) the results obtained for the IES‐27‐J can be considered as average to good. In making this comparison, however, it is important to note that sample characteristics such as the prevalence and mean severity of the disorder, as well as the number of items of the screening questionnaire, may influence the previously mentioned parameters (Terluin et al. 2020). In addition, the methodological limitations of the comparative studies (see Table S1), should be considered. Our results indicate that the IES‐27‐J can be applied validly as a diagnostic screening instrument for adolescents regarding the new BPD criteria as defined in ICD‐11.

### 4.1 Limitations and Strengths

Some methodological limitations of the present study should be considered. First, the diagnostic efficiency parameters such as SEN and SPE, as well as the optimal cutoff value, can be influenced by the prevalence rate of other mental disorders and the severity of BPD symptoms (Terluin et al. 2020). Hence, the applicability of the results to an outpatient setting or to the general population with a lower prevalence rate might be further studied. Second, the interviewers were child and adolescent psychotherapists in training, not experienced interviewers. Third, given the higher proportion of females in the BPD group, we cannot calculate any analyses to examine gender effects that may have occurred.

A strength of the present study is the evaluation of diagnostic efficiency and the determination of optimal cutoff values for different diagnostic thresholds, thus giving consideration to the ongoing debate on the clinical utility of existing cutoff values in the evaluation of BPD among adolescents (Kaess et al. 2017). Another strength is the large sample size compared to that of most similar studies, which avoids the risk of an inaccurate estimation of the reported indices. The sample in the present study included several personality disorders and other mental disorders, which are significant distractors for the IES‐27‐J. This made it possible to evaluate the instrument under conditions that place particular methodological demands on the IES‐27‐J. Since we used juveniles who were in outpatient psychiatric care and seeking treatment in an inpatient or day hospital unit, the ecological validity of the results can be assumed. The assessment of diagnostic efficiency was based on a comprehensive reporting of indices for different cutoffs, making the determination of the optimal cutoff transparent and comprehensible to the reader.

### 4.2 Implications and Future Research

An application of the IES‐27‐J in clinical practice could be the its use within a two‐stage diagnostic procedure. As a first step, a lower cutoff value could be chosen to miss fewer patients with BPD. In a second step, if the cutoff value is exceeded, a clinical interview could be conducted to confirm a possible diagnosis. Although this procedure would be very time‐consuming, it could be justified given the health‐related and economic consequences of failing to identify adolescents with BPD. To draw more valid conclusions on how to evaluate the diagnostic efficiency of the IES‐27‐J compared to other screening instruments, direct comparisons within one study would be desirable. In addition, the inclusion of adolescents without mental disorders in the validation of the questionnaire could provide further insight into the extent to which the IES‐27‐J is able to discriminate between adolescents with clinically relevant BPD and adolescents with impulsivity and emotional dysregulation in the context of normal adolescent development.

## 5 Conclusion

The IES‐27‐J again demonstrated good psychometric properties and high reliability indices. Diagnostic efficiency indices did not reach the recommended level. However, the application of the IES‐27‐J can be considered to be used in a two‐stage approach. This study contributes to ensure successful BPD‐specific diagnostics, allowing patients to be identified and treated at an early stage of the disorder.

## Author Contributions

**Maria Brede:** data curation, methodology, formal analysis, writing – original draft, writing – review and editing. **Brigitte Dippold:** supervision, validation. **Stephan Bender:** resources. **Christoph Kröger:** conceptualization, supervision, writing – review and editing, validation. **Maya krischer:** project administration, investigation. All authors read and approved the manuscript and contributed to critical review. Christoph Kröger and Maya Krischer share last authorship.

## Ethics Statement

The study has been approved by the Ethics Committee of the Medical Faculty of the University of Cologne (number 16‐122). Participation to the study was voluntary.

## Consent

All participants and at least one legal guardian gave written informed consent.

## Conflicts of Interest

The authors declare no conflicts of interest.

## Supporting information

Supporting information.

Supporting information.

Supporting information.

Supporting information.

Supporting information.

## Data Availability

This study was not preregistered. The data that support the findings of this study are openly available in the Open Science Framework at https://osf.io/7tqhs/?view_only=5fca0e040a494ac4bb3ee09dc924bd31.
