# Supplementary material for: Identification of Borderline Personality Disorder in Adolescents: Psychometric Properties and Diagnostic Efficiency of a Juvenile Version of the Impulsivity and Emotion Dysregulation Scale (IES‐27‐J)
Source: J Clin Psychol. 2025 Mar 25;81(7):567–76. doi: 10.1002/jclp.23792 (PMC12148295; doi:10.1002/jclp.23792)
Supplement: Supplementary file 1 — Supporting information. [file JCLP-81-567-s002.docx]

**Table S1**

*Overview of studies that have investigated the diagnostic efficiency of BPD-specific screening instruments for adolescents*

| Screening instrument | Number of items | Studies | Actual and recommended sample size | Basis rate of BPD and sample characteristics | Results | Methodological limitations |
| --- | --- | --- | --- | --- | --- | --- |
| Borderline Personality Disorder Features Scale for Children (BPFS-C; Crick et al., 2005) | 24 | Chang, Sharp, & Ha (2011) | *N* = 51  *N_recommended_* = 204^a^ | 39%  psychiatric inpatients  age 12 to 18 years | Cronbach’s α = .89  AUC = .93  SEN = .86  SPE = .84 | 1), 2), 3), 4), 5)^c^ |
| Borderline Personality Disorder Features Scale for Children – Short Version (BPFS-C-11) | 11 | Sharp et al. (2014) | *N* = 371  *N_recommended_* = 341^a^ | 33%  psychiatric inpatients  age not specified | Cronbach’s α = .85  AUC = .80  SEN = .74  SPE = .71 | 2), 3), 4), 5)^c^ |
| Borderline Personality Questionnaire (BPQ; Poreh et al., 2006) | 80 | Chanen et al. (2008) | *N* = 101  *N_recommended_* = 427^a^ | 22%  mental health service outpatients  age 15 to 25 years | Cronbach’s α = .92  AUC^b^ = .85  SEN = .68  SPE = .90  NPV = .91  PPV = .65  $\kappa$ = .57 with SCID-II | 1), 3), 4), 5)^c^  AUC-curve-based selection of optimal cutoff |
| McLean Screening Instrument for BPD (MSI-BPD; Zanarini et al., 2003) | 10 | Chanen et al. (2008)  Noblin et al. (2013) | *N* = 101  *N_recommended_* = 427^a^  *N* = 118  *N_recommended_* = 420^a^ | 22%  mental health service outpatients  age 15 to 25 years  26%  acute care inpatients  age 12 to 17 years | Cronbach’s α = .78  AUC^b^ = .73  SEN = .68  SPE = .75  NPV = .89  PPV = .43  $\kappa$ = .35 with SCID-II  Cronbach’s α = .73  AUC = .73  SEN = .71  SPE = .66 | 1), 3), 4), 5)^c^  AUC-curve-based selection of optimal cutoff  1), 2), 3), 5) |

*Notes.* ^a^ The recommended sample size was determined using the following formula from Negida et al. (2019) and Hajian-Tilaki (2014; formulas 6.6 and 6.7), with the maximum marginal error *d* set to 0.1.

$N= N_{se}+ N_{sp}=\frac{Z_{\frac{\alpha}{2}}^{2}\hat{Se}(1-\hat{Se})}{d^{2}\times Prev}+ \frac{Z_{\frac{\alpha}{2}}^{2}\hat{Sp}(1-\hat{Sp})}{d^{2}\times(1-Prev)}$ (Negida et al., 2019; Hajian-Tilaki, 2014)

^b^ The authors used the term “overall diagnostic accuracy”.

^c^ 1) Sample size smaller than recommended; results in increased marginal error, i.e., reduced precision of estimated diagnostic accuracy indices (Hajian-Tilaki, 2014). 2) No report of (weighted) κ and no cross-tables provided. 3) No weighting of sensitivity when selecting the optimal cut-off (Zimmerman & Ballig, 2019). 4) No report of indices for different cut-offs. 5) Cronbach’s α not corrected for commingled samples (Waller, 2008).
