# Supplementary material for: Identification of Borderline Personality Disorder in Adolescents: Psychometric Properties and Diagnostic Efficiency of a Juvenile Version of the Impulsivity and Emotion Dysregulation Scale (IES‐27‐J)
Source: J Clin Psychol. 2025 Mar 25;81(7):567–76. doi: 10.1002/jclp.23792 (PMC12148295; doi:10.1002/jclp.23792)
Supplement: Supplementary file 2 — Supporting information. [file JCLP-81-567-s004.docx]

**Calculation S2**

*Sample Size Calculation*

The recommended sample size was determined using the following formula from Negida et al. (2019) and Hajian-Tilaki (2014; formulas 6.6 and 6.7), with the maximum marginal error *d* set to 0.1, as proposed by Negida et al. (2019).

$N= N_{se}+ N_{sp}=\frac{Z_{\frac{\alpha}{2}}^{2}\hat{Se}(1-\hat{Se})}{d^{2}\times Prev}+ \frac{Z_{\frac{\alpha}{2}}^{2}\hat{Sp}(1-\hat{Sp})}{d^{2}\times(1-Prev)}$

Considering the parameters from Chang et al. (2011), this resulted in a required sample size of *N* ≥ 204:

$N_{total}= \frac{Z_{\frac{\alpha}{2}}^{2}\hat{Se}(1-\hat{Se})}{d^{2}\times Prev}+ \frac{Z_{\frac{\alpha}{2}}^{2}\hat{Sp}(1-\hat{Sp})}{d^{2}\times(1-Prev)}=\frac{{1.96}^{2}\times0.86 \times0.14}{{0.1}^{2}\times0.39}+ \frac{{1.96}^{2}\times0.84 \times0.16}{{0.1}^{2}\times0.61}=119+85=204$
