# Supplementary material for: Identification of Borderline Personality Disorder in Adolescents: Psychometric Properties and Diagnostic Efficiency of a Juvenile Version of the Impulsivity and Emotion Dysregulation Scale (IES‐27‐J)
Source: J Clin Psychol. 2025 Mar 25;81(7):567–76. doi: 10.1002/jclp.23792 (PMC12148295; doi:10.1002/jclp.23792)
Supplement: Supplementary file 3 — Supporting information. [file JCLP-81-567-s003.docx]

**Table S3**

*Frequency of personality disorders in accordance with the IPDE and DSM-IV*

| PD diagnoses: *n* (%) | Total sample  (*N* = 220) | BPD  (*n* = 88) | Other PDs and MDs  (*n* = 132) |
| --- | --- | --- | --- |
| Paranoid PD  Schizoid PD  Schizotypal PD  Antisocial PD  Histrionic PD  Narcissistic PD  Avoidant PD  Dependent PD  Obsessive-Compulsive PD  PD not otherwise specified | 17 (8%)  4 (2%)  3 (1%)  13 (6%)  9 (4%)  9 (4%)  98 (45%)  17 (8%)  11 (5%)  19 (9%) | 11 (13%)  1 (1%)  2 (2%)  7 (8%)  7 (8%)  4 (5%)  48 (55%)  8 (9%)  7 (8%)  1 (1%) | 6 (5%)  3 (2%)  1 (1%)  6 (5%)  2 (2%)  5 (4%)  50 (38%)  9 (7%)  4 (3%)  18 (14%) |

*Notes.* PD = Personality disorder. MD = Mental disorder.
