# Supplementary material for: Identification of Borderline Personality Disorder in Adolescents: Psychometric Properties and Diagnostic Efficiency of a Juvenile Version of the Impulsivity and Emotion Dysregulation Scale (IES‐27‐J)
Source: J Clin Psychol. 2025 Mar 25;81(7):567–76. doi: 10.1002/jclp.23792 (PMC12148295; doi:10.1002/jclp.23792)
Supplement: Supplementary file 5 — Supporting information. [file JCLP-81-567-s001.docx]

**Table S5**

*Contingency table of the classifications according to IPDE and IES-27-J for a cutoff value of ≥ 38*

|  | IPDE classification (five-criteria threshold) | | |  |
| --- | --- | --- | --- | --- |
| IES-27-J classification | | No BPD present | BPD present | Total |
| No BPD present | | 100 | 26 | 126 |
| BPS present | | 32 | 62 | 94 |
| Total | | 132 | 88 | 220 |
